# Effect of sodium–glucose transporter 2 inhibitor empagliflozin on proteinuria and kidney function progression in patients with non-diabetic glomerulonephritis: a pilot superiority randomized controlled trial

**DOI:** 10.1007/s11255-023-03539-8

**Published:** 2023-03-06

**Authors:** Hany Hammad, Asmaa Shaaban, Mariana Victor Philips, Ahmed Fayed, Tarek Samy Abdelaziz

**Affiliations:** 1 Nephrology Division—Department of Internal Medicine-Kasr-Alainy University Hospitals—Cairo University Hospitals, El Saray Street Manial, El Manial, Cairo, 11956 Egypt; 2 Department of Internal Medicine-Kasr-Alainy University Hospitals—Cairo University Hospitals, Cairo, Egypt

**Keywords:** Glomerulonephritis, Diabetes mellitus, Empagliflozin, Sodium–glucose transporter inhibitors, Proteinuria

## Abstract

**Background:**

Amelioration of proteinuria is one of main treatment targets in patients with glomerulonephritis, yet the remission rates are suboptimal.

**Aim of the study:**

To examine the effect of the sodium–glucose transporter 2 inhibitor (empagliflozin) on proteinuria and kidney function progression, in patients with glomerulonephritis not due to diabetic kidney diseases.

**Subjects and methods:**

Fifty patients were recruited. The entry criteria were diagnosis of glomerulonephritis, and proteinuria (proteinuria ≥ 500 mg⁄g) in spite of the use of the maximal tolerated dose of RAAS blocking agents together with specific immunosuppression treatment regimens. Group 1 (Empagliflozin arm): 25 patients who received 25 mg of empagliflozin once daily for 3 months as add-on to their regular treatment protocol (RAAS blockers and immunosuppression). Group 2 (Placebo arm): 25 patients treated with RAAS blockers and immunosuppression. The primary efficacy endpoints were the change in creatinine eGFR, and proteinuria 3 months after starting treatment.

**Results:**

Progression of proteinuria was lower with empagliflozin as compared to placebo (odds ratio, 0.65; 95% CI, 0.55 to 0.72, *p* = 0.002). Decline in eGFR was lower with empagliflozin as compared to placebo; however, this was statistically not significant (odds ratio, 0.84; 95% CI, 0.82 to 1.2, *p* = .31). The percentage change in proteinuria was greater with empagliflozin as compared to placebo (median, − 77 (− 97–105) vs − 48 (− 80–117).

**Conclusion:**

Empagliflozin has a favorable effect on amelioration of proteinuria in patients with glomerulonephritis. Empagliflozin has tendency to preserve kidney function in patients with glomerulonephritis as compared to placebo; however, longer term studies are required.

## Introduction

There are two glucose transporters that are important for glucose reabsorption; both are in the proximal tubules of the kidneys: sodium–glucose cotransporter 1 (SGLT1), located in the S3 segment of the proximal tubule, and sodium–glucose cotransporter 2 (SGLT2), located in the S1 segment of the renal proximal tubule. SGLT2 are responsible for reabsorption of about 90% of the filtered glucose [1].

Phlorizin (the precursor of all SGLT inhibitors) is an old natural compound derived from the bark of apple trees that was found to increase urinary glucose excretion [2]. It causes non-selective inhibition of both SGLT1 and SGLT2, causing gastrointestinal side effects (diarrhea and malabsorption) [3].

Sodium–glucose cotransporter-2 (SGLT2) inhibitors are novel class of oral antihyperglycemic medications. They exert antihyperglycemic effects by enhancing glucose excretion through renal tubules (glycosuria) through highly selective inhibition of SGLT2. The selectivity of this class markedly reduced gastrointestinal side effects that were associated with SGLT 1 inhibition [4].

In 2014, the U.S. Food and Drug Administration approved two agents of the SGLT2 inhibitors: dapagliflozin and empagliflozin [3]. Large clinical trials demonstrated that SGLT2 inhibitors (dapagliflozin, empagliflozin and canagliflozin) lead to improved cardiac and renal outcomes in patients with type 2 diabetes mellitus [5–9]. Different renal end points were assessed in major clinical trials, including progression to macro albuminuria, doubling of serum creatinine or reduction in estimated glomerular filtration rate (eGFR).

Interestingly, in addition to their glycosuria effect, these agents were found to have beneficial metabolic effects which included uricosuric, blood pressure lowering and weight reduction effects [10].

The beneficial effects of SGLT2 inhibitors on diabetic kidney diseases led to extending clinical trials to CKD patients due to causes other than diabetic kidney disease. The Dapagliflozin in Patients with Chronic Kidney Disease (DAPA-CKD) study was large phase 3 study to assess dapagliflozin in patients with CKD due to diabetes or any other cause. The study was powered for the renal outcomes. The progression to end-stage renal disease and death due to renal cause was lower in the dapagliflozin arm [11].

The Empagliflozin in Patients with Chronic Kidney Disease (EMPA-KIDNEY), of empagliflozin is a large clinical trial that is powered for the prespecified renal outcomes in CKD not due to diabetes mellitus. The use of Empagliflozin has been shown to preserve renal function as compared to placebo [12].

Glomerulonephritis (GN) describes a spectrum of inflammatory kidney disease. Advanced and treatment-resistant stages contribute to the burden of end stage renal disease [13, 14].

Primary glomerular diseases have wide spectrum of histopathological features including membranous nephropathy (MN), minimal change disease (MCD), focal segmental glomerulosclerosis (FSGS), IgA nephropathy and lupus nephritis (LN) [13]. The hallmark laboratory features of glomerulonephritis are proteinuria, hematuria, deranged kidney functions and hypoalbuminemia. Reducing proteinuria is one of the main therapeutic targets in patients with GN [15]. We have conducted this pilot randomized trial to examine the effect of add on treatment with empagliflozin on the amelioration of proteinuria in patients with glomerulonephritis.

## Methods

### Ethical approval

The study protocol was approved by Kasr-Alainy. Approval number is KA-21-297.

### Study design

This is a pilot randomized controlled trial.

### Sample size calculation

The sample size was calculated as 25 participants in each group to achieve 90% power with two-sided significance 5% and standardized effect size (0.5).

The pilot consisted of two arms.

### Empagliflozin arm

Included 25 patients with chronic glomerulonephritis who were prescribed 25 mg of empagliflozin once daily PO for 3 months in addition to their regular treatment protocol (RAAS blockers and immunosuppression).

### Placebo arm

Included 25 patients with chronic glomerulonephritis who were maintained on only RAAS blockers and immunosuppression

### Study registration

The study is registered to clinicaltrials.gov; registration number is NCT05283057.

### Study sites

The study was conducted at a single center which is tertiary care university hospital.

### Eligibility criteria

Eligible patients were in the age group ≥ 18 and ≤ 75 years and urinary protein excretion > 500 mg/g and eGFR ≥ 30 mL/min/1.73 m^2^ (CKD stages 1–3). They were required to be on stable doses of an ACEi or ARBs together with their immunosuppression protocol for at least 4 weeks prior to randomization. Women of Child-Bearing Potential (WOCBP) were required to use an acceptable method of contraception to avoid pregnancy throughout the study and for up to 4 weeks after the last dose of the study drug.

### Exclusion criteria

- Diagnosis of type 1 or type 2 diabetes mellitus
- Urinary protein excretion of less than 500 mg/g and eGFR < 30 ml/min/1.7 m^2^
- Active malignancy.
- Any medication, surgical or medical condition that may significantly alter the absorption, distribution, metabolism, or excretion of medications including, but not limited to any of the following: history of active inflammatory bowel disease within the last 6 months; Major gastrointestinal tract surgery such as gastrectomy, gastroenterostomy, or bowel resection; gastro-intestinal ulcers and/or gastrointestinal or rectal bleeding within last 6 months; pancreatic injury or pancreatitis within the last six months.
- Evidence of hepatic disease as determined by any one of the following: ALT or AST values exceeding 3 × ULN at the screening visit, a history of hepatic encephalopathy, a history of esophageal varices, or a history of portocaval shunt.
- Evidence of urinary tract obstruction.
- History of hypersensitivity or contraindications to empagliflozin.
- Subjects who, in the assessment of the investigator, may be at risk for dehydration or volume depletion that may affect the interpretation of efficacy or safety data.
- Participation in any clinical investigation within 3 months prior to initial dosing.
- Pregnancy or breastfeeding.

Laboratory workup included serum creatinine, estimated GFR by CKD-EPI creatinine equation, serum uric acid, serum albumin, full blood count, urine analysis, urinary protein to creatinine ratio and hemoglobin A1c.

### Randomization

Randomization was carried out using computer system to randomly assign each patient to one of the two arms.

### Intervention arm

Twenty-five patients received 25 mg of empagliflozin once daily PO for 3 months in addition to their regular treatment which included maximal tolerated dose of renin–angiotensin aldosterone system (RAAS) blockers and immunosuppressive medications

### Placebo arm

Twenty-five patients received placebo once daily PO for 3 months in addition to their regular treatment which included maximal tolerated dose of renin–angiotensin aldosterone system RAAS blockers and immunosuppressive medications

### Primary efficacy endpoints

The primary efficacy endpoint was the change in the level of proteinuria as measured by protein to creatinine ratio and the change in kidney function as measured by serum creatinine and eGFR.

### Statistical analysis

Data were collected and coded. Data were analyzed using IBM Statistical Package for Social Sciences software (SPSS), 21st edition, IBM, United States. Continuous data were expressed as mean ± standard deviation and categorical data as numbers and percentage. *T* test was used to compare the two groups. Quantitative data were expressed as mean and standard deviation. For comparisons between more than two groups, analysis of variance (ANOVA) was used. Chi-squared or Fisher’s Exact tests were used to compare between the qualitative data expressed as number and percentage. Correlation (Spearman and/or Pearson) were used to identify relations between data. Results are considered statistically significant at a *p* value of less than or equal 0.05. We used cox regression models to estimate hazard ratios, confidence intervals and *p* values.

## Results

### Baseline patients’ characteristics

Table 1 shows baseline patients characteristics. There were no significant differences between both groups as regards age or sex. The most common causes of proteinuria were minimal change disease, membranous nephropathy, and systemic lupus erythematosus. Causes of proteinuria were comparable across two groups.

**Table 1:** Baseline patient characteristics

		Empagliflozin		Control		*P* value
Age (mean SD)		32.72± 15.95		38.44± 17.33		0.56
Sex female no (%)		47%		51%		
		Count	%	Count	%	
HTN	Yes	12	48.0%	9	36.0%	0.390
Causes of glomerulonephritis	Alport disease	1	4.0%	1	4.0%	0.988
	FMF, Renal amyloidosis	1	4.0%	0	0.0%	
	Focal proliferative GN	1	4.0%	0	0.0%	
	FSGS	3	12.0%	5	20.0%	
	HCV MCGN	2	8.0%	2	8.0%	
	SLE	6	24%	4	16%	
	MCD	5	20.0%	5	20.0%	
	MN	4	16.0%	5	20.0%	
	Post streptococcal GN	1	4.0%	1	4.0%	
	Renal amyloidosis	0	0.0%	1	4.0%	
	Vasculitis, GPA	1	4.0%	1	4.0%	

There was no statistically significant difference between pretreatment values of both groups, as regards baseline protein to creatinine ratio, median serum creatinine and median eGFR (Table 2).

**Table 2:** Comparison of pretreatment values between both groups

	Empagliflozin			Placebo			*P* value
	Median	Min	Maximum	Median	Minimum	Maximum	
*S. creatinine* (pretreatment)	1.10	0.50	2.40	1.20	0.70	3.90	0.330
e GFR (pretreatment)	66.00	30	130	61.00	17.00	139.00	0.793
*S. albumin* (pretreatment)	3.20	1.90	4.60	3.60	2.20	5.10	0.075
Urine PCR (pretreatment)	2050	940	6870	2700	1099	5623.00	0.415

**Fig. 1:**
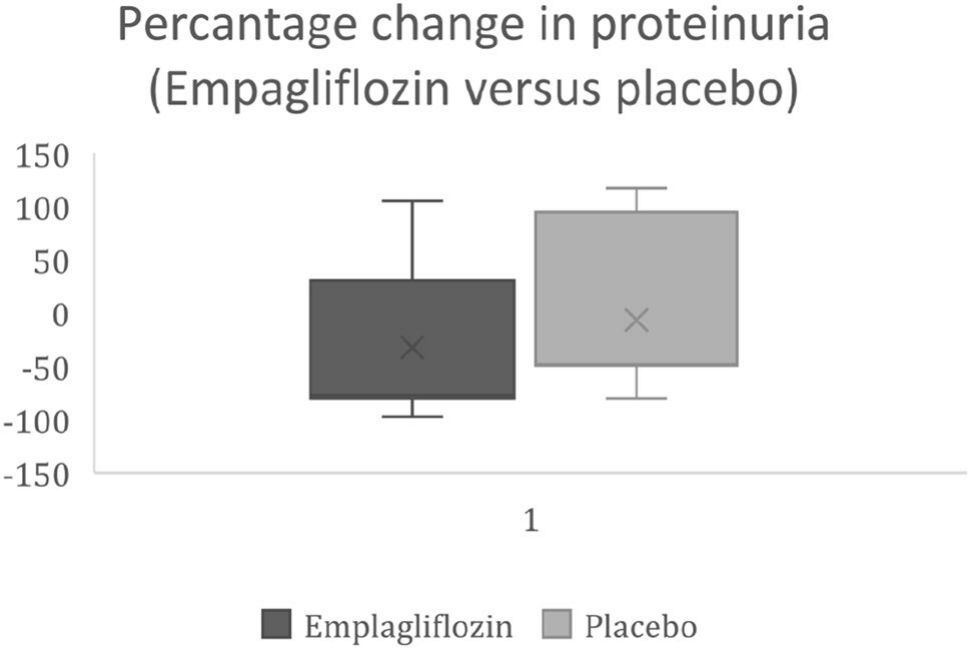
Percentage change of proteinuria after treatment (empagliflozin vs placebo)

The post-treatment values for the renal end points are shown in (Table 3). There was statistically significant difference between the empagliflozin group and the Placebo group as regard serum creatine, eGFR and urinary protein to creatinine ratio (Fig. 1).

**Table 3:** Comparison of the renal end points after 3 months of treatment (Empagliflozin vs Placebo)

	Empagliflozin			Placebo			*P* value
	Median	Minimum	Maximum	Median	Minimum	Maximum	
Creatinine (after 3 months)	0.90	0.5	1.60	1.30	0.70	5.10	0.007
e GFR (after 3 months)	88.00	41	147.00	56.00	11.00	146.00	0.05
Urine PCR (after 3 months)	556	72	1215.00	1598	545	3199.00	< 0.001

**Fig. 2:**
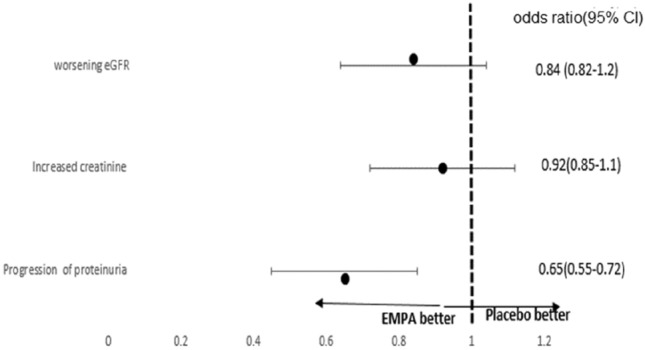
Odds ratio of renal endpoints with empagliflozin vs placebo

The use of empagliflozin was associated with more reduction of proteinuria as compared to placebo (Table 4). Percentage change in urinary protein to creatinine ratio, median (range)% change in empagliflozin group was (− 80.9 (− 97 to 105) compared to (− 48 (− 80 to 117) in the placebo group (*p* value < 0.001) (Fig. 2). Empagliflozin was associated with more preservation of kidney functions, as measured by eGFR and serum creatinine, However, this was statistically non-significant (e GFR % change (Median (interquartile range) 19.5 (− 42.2 to 51.7) in the empagliflozin group compared to 5.8 (− 46.3 to 35.3) in the placebo group, *p* = 0.078. The hazard ration for progression of proteinuria was lower in empagliflozin group as compared to place (Fig. 1).

**Table 4:** percentage changes in the renal end point

	Empagliflozin			Control			*P* value
	Median	Minimum	Maximum	Median	Minimum	Maximum	
Serum create. % change	− 14.29	− 60.87	18.18	− 6.25	− 46.15	75.00	0.076
e GFR % change	19.05	− 18.18	210.00	5.80	− 49.09	105.88	0.098
Urine PCR % change	− 77	− 97	105	− 48.07	− 80.56	117	< 0.001

Progression of proteinuria was lower with empagliflozin as compared to placebo (hazard ratio, 0.65; 95% CI 0.55–0.72, *p* value 0.002). Decline in eGFR was lower with empagliflozin as compared to placebo; however, this was statistically not significant (odds ratio, 0.84; 95% CI 0.82–1.2, *p* value 0.31). The percentage change in proteinuria was more with empagliflozin as compared to placebo (median, − 77 (− 97 to 105) vs − 48 (− 80 to 117).

## Discussion

In this pilot randomized controlled trial, we assessed the effect of empagliflozin on urine protein excretion and preservation of kidney function as measured eGFR, in patients with non-diabetic glomerulonephritis. Empagliflozin 25 mg once daily efficacy was tested against standard treatment (RAAS blockers and immunosuppression) alone (placebo group). There were no significant differences between both groups as age and sex distribution.

Causes of glomerulonephritis were comparable in both groups. The most common causes of proteinuria in our cohort were MCD, FSGS, lupus nephritis and HCV-related glomerulonephritis.

The use of empagliflozin at a dose of 25 mg once daily, resulted in marked improvement in proteinuria as compared to the placebo (ACEi or ARBs plus immunosuppression alone. Empagliflozin had a favorable effect on preservation of kidney functions (eGFR and serum creatinine) as compared to placebo. However, this was statistically non-significant.

Reduction of proteinuria and preservation of kidney function are the two main goals of treatment of chronic glomerulonephritis. Sodium–glucose co-transporter 2 (SGLT2) inhibitors were successfully endorsed by guidelines as add on therapy in the management type 2 Diabetes mellitus. In large clinical trials, SGLT inhibitors demonstrated favorable effects on preservation of kidney function in patients with CKD.

There were no reported cases of genital infections, related to the use of empagliflozin in our cohort. some cases of asymptomatic bacteriuria were reported before and after treatment with no statistical significance. Previous studies have shown that the prevalence of urinary tract infections in patients treated with empagliflozin was not higher than control groups [16, 17].

Large multicenter studies have shown the favorable effects of the SGLT inhibiting agents on albuminuria and kidney function preservation. In the EMPA-REG OUTCOME trial, empagliflozin 10–25 mg/day reduced the incidence of the traditional renal composite outcome of doubling of creatinine by 46% [6]. Results of DAPA-CKD indicated a clear benefit in CKD management. DAPA-CKD trial enrolled a dedicated CKD population, and conclusively shows a benefit in this patient population, irrespective of DM status [11].

In a subgroup analysis of the DAPA-CKD trial, dapagliflozin use in patients with IgA nephropathy was associated with favorable effect on eGFR [11].

There were no hypoglycemic episodes reported in our cohort. Mean random blood glucose (RBG) level among the empagliflozin treated group was 91.64 ± 9.54 mg/dl before treatment versus 91.56 ± 8.13 mg/dl after treatment, with no statistically significant difference (*p* = 1). In our study empagliflozin treatment was associated with lower serum uric acid as compared to placebo (*p* value < 0.001). The hypouricemic effect of SGLT inhibitors is well established as a class effect of SGLT inhibitors. This has been shown in previous clinical trials and meta-analysis [18]**.**

The strength of our study is that it is one of the earliest studies to assess empagliflozin in patient with glomerulonephritis other than diabetic kidney disease.

### Limitations

The study has some limitations. First, the study was a single-centered study. Ideally, a multi-center study will provide more information. Second, the recruited numbers are small. However, being a pilot study, it will help inform the effect size and standardized mean of difference for future randomized trial.

## Conclusion

In this pilot study, empagliflozin has a favorable effect on amelioration of proteinuria in patients with glomerulonephritis. Empagliflozin has tendency to preserve kidney function progression in patients with glomerulonephritis as compared to placebo; however, larger randomized studies are required.
